# Photosensitivity-Induced Pediatric Rash Following Surgery: A Case Study

**DOI:** 10.7759/cureus.69529

**Published:** 2024-09-16

**Authors:** Zaid Almubaid, Zachrieh Alhaj, Guillermo Saldana, Osvaldo Alquicira, Sharif Mohamed

**Affiliations:** 1 Medicine, John Sealy School of Medicine, University of Texas Medical Branch, Galveston, USA; 2 Anesthesiology, Tilman J. Fertitta Family College of Medicine, Houston, USA; 3 Anesthesiology, University of Texas Medical Branch, Galveston, USA

**Keywords:** anesthesiology, led bulb, pediatric derm, pediatrics medicine, skin photosensitivity

## Abstract

Photosensitivity is characterized by an abnormal skin reaction to light that causes various adverse skin reactions such as blistering, redness, and inflammation. Photosensitivity may also depend on the wavelength of the light as minor changes affect different layers of the skin. During surgical procedures, many different types of light are used with a range of wavelengths. Some of these illumination sources include light-emitting diode lights, xenon light, and many others. This case study examines the occurrence of a photosensitivity reaction in a patient by considering the length, duration, and type of light during surgery.

Our patient is a four-year-old male who presented to University of Texas Medical Branch at Galveston (UTMB) with phimosis and umbilical hernia. The patient underwent umbilical herniorrhaphy and circumcision. The patient received general anesthesia via inhalation for both induction and maintenance. The patient's vitals were stable before and after performing the procedure. After completion of the procedure, the patient appeared to have a red rash along the left side of his upper thigh and lower groin area. There were no other complications from the surgery and the procedure was successful. Thus, it was presumed as an erythematous skin burn caused by the operating room (OR) lights.

The findings in this case demonstrate a novel finding of a sunburn-like rash caused by the OR lights. Further research is indicated to determine other risk factors such as the type of light being used in the OR, the distance of the light to the exposed skin (which may vary based on surgeon preference), and the duration of time that the lights were used.

## Introduction

Photosensitivity is characterized by an abnormal skin reaction to light that causes various adverse skin reactions such as blistering, redness, and inflammation [1]. These reactions are often associated with the use of photosensitizing drugs but may also occur due to an allergy or direct ultraviolet wave exposure [1]. Genetic disorders such as erythropoietic protoporphyria and xeroderma pigmentosum may also lead to photosensitive skin [2,3].

Photosensitivity may also depend on the wavelength of the light as minor changes affect different layers of the skin [4]. For example, visible and infrared light can penetrate the dermis, causing damage and matrix degradation [5]. During surgical procedures, many different types of light are used with a range of wavelengths. Some of these illumination sources include light-emitting diode lights, xenon light, and many others [6,7]. Burn risks in the operating room (OR) may depend on many other factors such as the duration of light exposure and distance from the illuminating source. Another factor that could modify sensitivity is the concentration of melanin. Skin with lighter complexion for example has more UV damage than skin with a darker complexion, where the melanin acts as a protective factor [8].

This case study examines the occurrence of a photosensitivity reaction on a patient by considering the length, duration, and type of light during surgery. It illustrates the importance of developing preventative measures to avoid these types of skin reactions. Additionally, it emphasizes the need for further research on light-induced burns in the OR.

## Case presentation

Our case examines a four-year-old male who was hospitalized at the University of Texas Medical Branch (UTMB) for phimosis and an umbilical hernia, scheduled to undergo an umbilical herniorrhaphy and circumcision. The patient underwent general anesthesia via inhalation for both induction and maintenance and was stable before, during, and after the procedures. However, after completion of the procedures, the patient presented with a red rash along his left upper thigh and lower groin area within hours after the procedure. 

**Figure 1:**
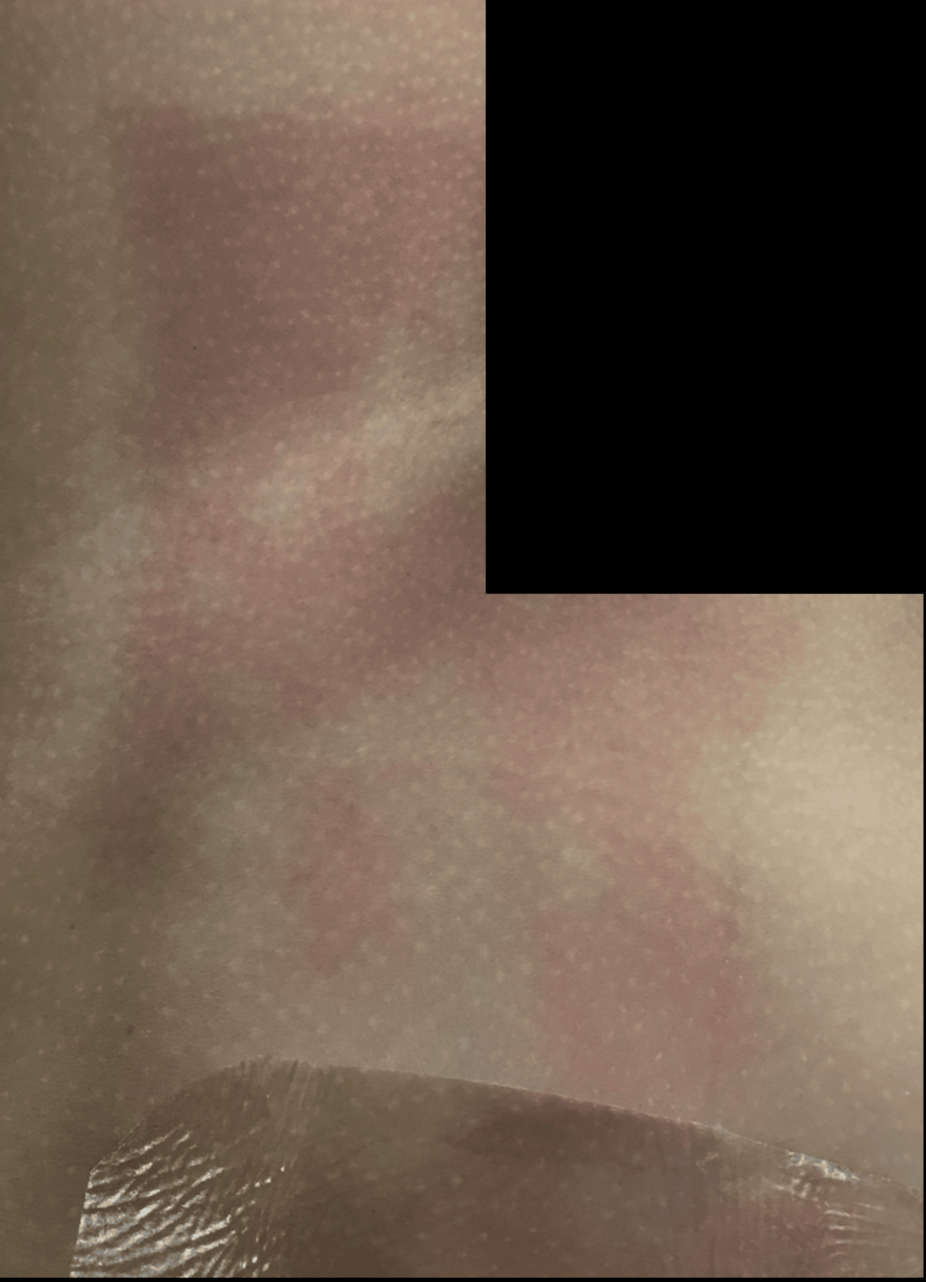
Picture of the rash on the patient post-surgery

As depicted in Figure 1, clear demarcations can be seen showing the area exposed to the OR light, characterized as a flat, well-bordered erythematous rash, as opposed to the area unaffected skin covered by the surgical drapes. We hypothesized the rash was a photosensitivity rash from the OR lights because the rash was in the areas exposed to light during the procedure. Although dermatological reactions to OR lights are rare, they can occur, and they usually manifest as erythematous rashes in exposed areas due to prolonged light wavelength exposure [9].

While the outcomes of this procedure were favorable, the occurrence of the rash demonstrates the importance of consideration of all environmental factors within the OR. Further evaluation and potential modifications in lighting protocols or protective measures may be warranted to prevent similar occurrences in future cases. Some possible modifications that can occur are reducing the intensity of OR lights, applying skin protectants and draping patients, and limiting patient exposure time to light.

## Discussion

This case report assesses the occurrence of a rash, induced by OR light exposure. While the procedure performed on this patient was successful and the patient recovered without complications, the incident emphasizes the importance of environmental factors, such as lighting, in surgical settings. Healthcare providers must remain vigilant in monitoring patients for unexpected reactions and implementing preventive measures to minimize potential risks.

Photosensitivity reactions in the OR can be attributed to several factors, including the type of light used, its intensity, distance from the patient, and the duration of exposure. The intensity of light and its wavelength can cause skin damage through mechanisms such as increased oxidative stress, cellular damage, and apoptosis; this, in turn, can result in erythema and rashes [10]. The type of light used, its intensity, distance from the patient, and duration of exposure are all factors that increase the risk of light-induced skin reactions [11]. Pediatric skin is known to be more sensitive and susceptible to various irritants, including light-induced reactions [12]. The light used in OR settings can penetrate the skin more deeply and cause more significant damage in children compared to adults. Therefore, adjusting the lighting protocols in the OR to minimize these risks is essential.

Management of photosensitivity reactions in surgical settings can be done in various ways. For starters, using lights with adjustable intensity and directing the light away from sensitive areas can help reduce the risk. Also, applying physical barriers, such as surgical drapes or protective covers, can shield the patient's skin from direct light exposure. If a photosensitivity rash occurs, treatment typically involves supportive care [13]. This may include the use of topical corticosteroids to reduce inflammation and emollients to protect the skin [13]. 

Future studies should be done on factors that can be adjusted to reduce chances of these risks. These may include things such as the type of OR light used in the case, the distance of the light from the skin, and the duration of exposure to the light that was used. 

## Conclusions

The findings in this case demonstrate a photosensitivity rash caused by the OR lights. Further research is indicated to determine other risk factors such as the type of light being used in the OR, the distance of the light to the exposed skin (which may vary based on surgeon preference), and the duration of time that the lights were used. Moving forward, further investigation into lighting protocols and protective measures in the OR could help prevent similar occurrences in pediatric patients.

Additionally, this case should encourage the field of anesthesia to address the environmental factors in the OR that increase the chances of skin irritation. Working with other professionals can help aid the development of strategies to minimize light-induced skin reactions. By addressing these concerns, all aspects of patient care can be enhanced for the benefit of patients. Future practices should reflect these changes to ensure a safer OR environment for all patients.

## References

[1] Montgomery S, Worswick S (2022). Photosensitizing drug reactions. Clin Dermatol.

[2] Lecha M, Puy H, Deybach JC (2009). Erythropoietic protoporphyria. Orphanet J Rare Dis.

[3] Moriwaki S, Kanda F, Hayashi M, Yamashita D, Sakai Y, Nishigori C (2017). Xeroderma pigmentosum clinical practice guidelines. J Dermatol.

[4] Meinhardt M, Krebs R, Anders A, Heinrich U, Tronnier H (2008). Wavelength-dependent penetration depths of ultraviolet radiation in human skin. J Biomed Opt.

[5] Salsberg J, Andriessen A, Abdulla S, Ahluwalia R, Beecker J, Sander M, Schachter J (2019). A review of protection against exposome factors impacting facial skin barrier function with 89% mineralizing thermal water. J Cosmet Dermatol.

[6] Knulst AJ, Stassen LP, Grimbergen CA, Dankelman J (2009). Choosing surgical lighting in the LED era. Surg Innov.

[7] Das A, Mitra S, Agarwal P, Sengupta A (2020). Prolonged intra-operative thermal exposure in endoscopic ear surgery: is it really safe?. J Laryngol Otol.

[8] Yamaguchi Y, Takahashi K, Zmudzka BZ (2006). Human skin responses to UV radiation: pigment in the upper epidermis protects against DNA damage in the lower epidermis and facilitates apoptosis. FASEB J.

[9] Surmeli-Onay O, Korkmaz A, Yigit S, Yurdakok M (2013). Phototherapy rash in newborn infants: does it differ between conventional and light emitting diode phototherapy?. Pediatr Dermatol.

[10] Svobodova A, Walterova D, Vostalova J (2006). Ultraviolet light induced alteration to the skin. Biomed Pap Med Fac Univ Palacky Olomouc Czech Repub.

[11] (2020). Light-emitting diodes (LEDS): implications for safety. Health Phys.

[12] Berardesca E, Farage M, Maibach H (2013). Sensitive skin: an overview. Int J Cosmet Sci.

[13] (2019). Dermatologic Reactions to Cancer Therapies. https://doi.org/10.1201/9781315267364.

